# La relation entre la récurrence de la tumeur et les polymorphismes génétiques de hGPX1 et NRAMP1 chez les patients atteints du cancer superficiel de la vessie: une méta-analyse

**DOI:** 10.11604/pamj.2017.27.270.12282

**Published:** 2017-08-10

**Authors:** Tapara Dramani Maman Souraka, Ming-Jun Shi, Xiang-Yu Meng

**Affiliations:** 1 Département de Diagnostic Génétique, Hôpital Zhongnan de l’Université de Wuhan, 169 Rue Donghu, Wuchang, Wuhan 430071, Chine; 2 Institut Curie, PSL Research University, CNRS, UMR 144, F-75005, Paris, France; 3 Université Paris Sud, Université Paris-Saclay, CNRS, UMR 144, F-91405 Orsay, France; 4 Centre de Médecine Fondée sur les Preuves et de Médecine Translationelle, Hôpital Zhongnan de l'Université de Wuhan, 169 Rue Donghu, Wuchang, Wuhan 430071, Chine; 5 Département de Médecine Fondée sur les Preuves et d’Epidémiologie Clinique, Deuxième École de Médecine Clinique de l’Université de Wuhan, 169 Rue Donghu, Wuchang, Wuhan 430071, Chine

**Keywords:** NRAMP1 D534N, hGPX1 Pro168Leu, NMIBC, récurrence, méta-analyse, NRAMP1 D534N, hGPX1 Pro168Leu, NMIBC, recurrence, meta-analysis

## Abstract

**Introduction:**

Des études antérieures ont fait preuves de résultats incohérents concernant la relation entre la récurrence et les polymorphismes génétiques de hGPX1 et NRAMP1 chez les patients NMBIC.

**Méthodes:**

On a effectuée une recherche bibliographique systématique sur la base de données de PubMed et de China National Knowledge Infrastructure. Selon des critères de sélection prédéfinis, l'éligibilité des études retrouvés a été évaluée par deux auteurs indépendants. Les caractéristiques basiques des études incluses et données pertinentes pour la méta-analyse sont extraites. La survie sans récidive est choisie comme la mesure d'effet de méta-analyse.

**Résultats:**

Quatre publications sont retenues. Trois études ont évalué le NRAMP1 D534N, et trois le hGPX1 Pro168Leu. En fonction de l'association entre le NRAMP1 D534N et la récurrence tumorale, la méta-analyse n'a révélé aucune hétérogénéité significative, et l'effet combiné est 3,28 (1,77- 6,11). En fonction de l'association entre le hGPX1 Pro198Leu et la réccurence tumorale, la méta-analyse a révélé une hétérogénéité significative, et l'effet combiné est 1,12 (0,45- 2,77). Le biais de publication est incertaine en raison du nombre limité d'études incluses. L'instabilité des effets combiné est notée.

**Conclusion:**

Très peu de données sont disponibles concernant l'association entre la récurrence tumorale et les polymorphismes de NRAMP1 D534N et hGPX1 Pro168Leu chez les patients NMIBC. Le NRAMP1 D534N pourrait augmenter le risque de récurrence, mais l'effet du hGPX1 Pro168Leu n'est pas clair. Il serait souhaitable qu'une enquête plus approfondie soit faite sur une taille d'échantillon plus élevée afin de mieux expliquer ce phénomène.

## Abstract (en)

**Introduction:**

Previous studies about the relationship between tumor recurrence and NRAMP1 and HGPX1 gene polymorphism in patients with non-muscle-invasive bladder cancer (NMIBC) showed inconsistent results.

**Methods:**

We conducted a systematic review of the literature on the basis of data from PubMed and China National Knowledge Infrastructure. According to the predefined selection criteria, the eligibility criteria for the studies found in the literature were assessed by two independent authors. The basic characteristics of the included studies and of data relevant to the meta-analysis were extracted. Patients? survival without recurrence was selected as the measure of effect of meta-analysis.

**Results:**

Four publications were retained. Three studies evaluated the NRAMP1 D534N and three the hGPX1 Pro168Leu. Depending on the association between NRAMP1 D534N and tumor recurrence, the meta-analysis revealed no significant heterogeneity and the combined effect was 3.28 [1.77, 6.11]. Depending on the association between hGPX1 Pro198Leu and tumor recurrence, the meta-analysis showed significant heterogeneity and the combined effect was 1.12 [0.45, 2.77]. Publication bias was uncertain, due to the limited number of included studies.The instability of the combined effects was reported.

**Conclusion:**

Very little data are available on the association between tumor recurrence and NRAMP1 D534N and hGPX1 Pro168Leu poolymorphisms in patients with NMIBC. The NRAMP1 D534N could increase the risk of recurrence, but hGPX1 Pro168Leu effect is not clear. A more thorough investigation should be conducted on a larger sample size in order to better explain this phenomenon.

## Introduction

La récurrence tumorale est fréquemment observée chez des patients atteints de cancer de la vessie non invasifs (NMIBC) [1]. En plus de certaines variables cliniques et pathologiques, la valeur prédictive de plusieurs biomarqueurs a été étudiée dans des études antérieures, y compris des facteurs de susceptibilité génétique [2, 3]. Par example, concernant cette question, les polymorphismes des gènes impliqués dans la désintoxication métabolique et la susceptibilité à la tuberculose ont été examinés [4, 5]. Le human glutathione peroxidase 1 (hGPX1), une enzyme dépendante du sélénium, est reconnue associée à la désintoxication du peroxyde d'hydrogène, des peroxydes organiques et des radicaux oxydants liés au tabagisme [6–8]. Selon Zhao et al., une substitution nucléotidique au codon 198 (C > T) qui entraîne la proline remplacée par la leucine (Pro198Leu), est protectrice en cas de récurrence [4]. Cependant, Chiong et al. a soutenu que cette mutation est un indicateur négatif de survie sans récidive (SSR) chez les patients traités par le bacillus Calmette-Guérin (BCG) [9]. Des résultats incohérents sont aussi rapportés dans les études portant sur le D543N (G > A) polymorphisme de natural resistance-associated macrophage protein 1 (NRAMP1) [5, 9, 10]. Pour cette raison, on a effectué cette synthèse méthodique, pour résumer les données disponibles et obtenir une conclusion globale plus précise.

## Méthodes

**Recherche bibliographique et sélection d'études:** On a effectué une recherche bibliographique systématique sur la base de données de PubMed et China National Knowledge Infrastructure (CNKI). La dernière recherche a été effectuée le 15 janvier 2017. Les termes de recherche suivants et les termes chinois correspondants ont été utilisés : “natural resistance-associated macrophage protein 1 or NRAMP1”, “human glutathione peroxidase 1 or hGPX1”, “polymorphism or variant or mutant”, et “bladder cancer”. Les études répondant aux critères suivants ont été incluses : étude d'essai clinique ou cohorte ; la relation du NRAMP1 D534N et/ou hGPX1 Pro168Leu avec la récurrence de NMIBC a été évalué ; hazard ratio (HR) et intervalle de confiance à 95 pour cent (IC à 95%) ont été calculés pour la SSR.

**Extraction de données:** Les données des études éligibles ont été recueillies de manière indépendante par deux auteurs (MXY et SMJ). Tout désaccord fut par la suite résolu par la discussion avec un troisième auteur (TDMS) jusqu´à ce que le consensus soit obtenu. Les informations suivantes ont été extraites de chaque article: le premier auteur, nombre total de patients, origine ethnique, traitement, méthode de génotypage, distribution du génotype pour chaque polymorphisme, et données pour la méta-analyse (HR et IC à 95%).

**Méta-analyse:** La méthode statistique de l'I-carré et le Q test ont été utilisés pour évaluer l'hétérogénéité des études incluses. Si l'I-carré était moins de 50% et P du Q test pas moins de 0,1, le modèle à effets fixes serait utilisé car aucune hétérogénéité significative était detectée; dans d'autres cas où une hétérogénéité significative était notée, le modèle à effets aléatoires serait utilisé [11]. L'IC à 95% des effets combinés ne traversant pas 1 signifie une association significative. De plus, on a effectué une analyse de sensibilité pour tester la stabilité des effets combinés, et chaque fois on a exclu une seule étude. Les graphiques en forêt ont été générés et les graphiques en entonnoir ont été utilisés pour éxaminer le biais de publication [12]. Des analyses ont été effectuées à l'aide du logiciel Review Manange 5.3 (Oxford, Angleterre, Royaume-Uni).

## Résultats

**Figure 1:**
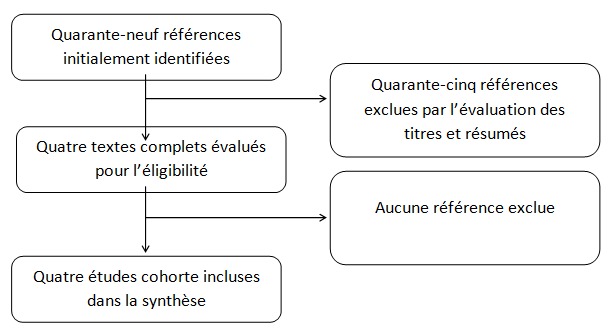
Organigramme de la recherche bibliographique et selection d’études

**Recherche bibliographique et inclusion d'études:** La recherche initiale a identifié quarante-neuf références, dont quarante-cinq ont été exclues après l'examination du titre et résumé [4, 5, 9, 10]. Après la vérification du texte complet, quatre études ont été finalement retenues. Trois études ont examiné les polymorphismes de NRAMP1 [5, 9, 10], et trois études ont examiné ceux de hGPX1 [4, 9, 10]. L'organigramme de la recherche bibliographique et de la selection d'études est montré sur la Figure 1. Les caractéristiques basiques des études incluses sont presentées dans le Tableau 1L

**Tableau 1:** Caractéristiques basiques des études incluses

Premier auteur	Année de publication	Nombre total de patients	Origine ethnique	Traitement	Méthode de génotypage	Distribution du génotype NRAMP1	Distribution du génotype hGPX1
Zhao H. [4]	2005	224	Caucasien(202) Autres(22)	BCG(119) Autres(105)	PCR	Indisponible	CC(116) CT(104) TT(4)
Decobert M. [5]	2006	67	Indisponible	BCG(67)	PCR	GG(59) GA(8)	Indisponible
Chiong E. [9]	2011	99	Asiastique(97) Caucasien(2)	BCG(99)	PCR	GG(67) GA(32)	CC(93) CT(6)
Lenormand C.[10]	2016	92	Indisponible	BCG(92)	PCR	GG(88) GA(4)	CC(52) CT(35) TT(5)

PCR : réaction en chaîne par polymérase. BCG : bacillus Calmette-Guérin

**Figure 2:**
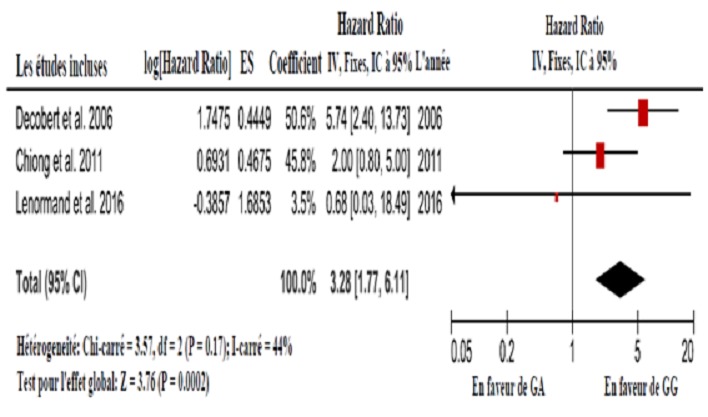
Graphique en forêt montrant la méta-analyse pour NARMP1 D534N

**Figure 3:**
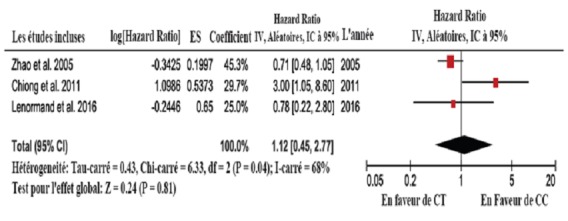
Graphique en forêt montrant la méta-analyse pour hGPX1 Pro168Leu

**Méta-analyse:** En fonction de l'association entre le NRAMP1 D534N et la récurrence tumorale, la méta-analyse n'a révélé aucune hétérogénéité significative (I-carré = 44%, P = 0.17) et le modèle à effets fixes a été utilisé. L'HR et IC à 95% (GA vs. GG) combiné est 3.28 ((1.77, 6.11)(P = 0.0002)). Cela signifie qu'il y a un effet global statistiquement significatif; en d´autres termes, l'allèle muté augmente le risque de récurrence. Le graphique en forêt est présenté sur la Figure 2. En raison du nombre limité d'études incluses, il est difficile de déterminer la symétrie du graphique en entonoir (pas montré), donc l'existence d'un biais de publication est incertaine. En fonction de l'association entre le hGPX1 Pro198Leu et la récurrence tumorale, la méta-analyse a révélé une hétérogénéité significative (I-carré = 68%, P = 0.04) et le modèle à effets aléatoires a été utilisé. L'HR et IC à 95% (CT vs. CC) combiné est 1.12 ((0.45, 2.77) (P = 0.81)). Cela signifie qu'il y a un effet global statistiquement non significatif. L'incertitude concernant la relation entre ce polymorphisme et le risque de récurrence demeure. Le graphique en forêt est présenté sur la Figure 3. En raison du nombre limité d´études incluses, il est difficile de déterminer la symétrie du graphique en entonoir (pas montré), donc l'existence d'un biais de publication est incertaine.

**Analyse de sensibilité:** Une grande variation a été observée dans l'analyse de sensibilité, qui signifie l'instabilité des effets combinés. Les résultats sont présentés dans le Tableau 2.

**Tableau 2:** Résultats de l’analyse de sensibilité

Opération	NRAMP1 D534NGA vs. GG	hGPX1 Pro168Leu GA vs. GG
Global	3,28(177- 6,11)	1,12(0,45- 2,77)
À l'exclusion de Zhao et al. [4]	N.A.	1,61(0,43- 5,99)
À l'exclusion de Decobert et al. [5]	1,85(0,77- 4,48)	N.A.
À l'exclusion de Chiong et al. [9]	5,00(2,15- 11,61)	0,72(0,49- 1,04)
À l'exclusion de Lenormand et al. [10]	3,48(1,85- 6,54)	1,34(0,33- 5,44)

N.A. : N’est pas applicable.

## Discussion

La tumeur papillaire superficielle est la forme la plus courante du cancer de la vessie, et son traitement primaire est la résection transurétrale [13, 14]. La récurrence est fréquente et l'instillation intravésicale de BCG et/ou d'agents de chimiothérapie est la norme actuelle pour prévenir la récurrence [15]. Sur ce sujet, des études antérieures ont étudié le rôle de plusieurs polymorphismes génétiques, parmi lesquels le NRAMP1 D534N et le hGPX1 Pro168Leu ont reçu une attention [4, 5, 9, 10]. La première littérature pertinente a été publiée en 2005, dans laquelle Zhao et al. ont affirmé que la variante T au codon 198 de hGPX1 protège contre la récurrence [4]. L'année suivante, Decobert et al. ont mesuré la relation entre NRAMP1 D543N et la récurrence dans une série de 67 patients NMIBC, et l'allèle A était considéré comme nuisible en ce qui concerne la récurrence [5]. En 2011, Chiong et al. ont publié une étude approfondie et différents résultats ont été rapportés. Selon leurs résultats, le hGPX1 Pro168Leu était significativement et négativement associé avec le SSR, mais la relation entre le NRAMP1 D534N et le SSR n'était pas significative, chez les patients NMIBC traités par le BCG [9]. La plus récente publication de Lenormand et al. n'a trouvé aucune association significative [10]. Afin de résoudre l'évidente discordance entre les études individuelles, nous avons réalisé cette méta-analyse. Selon le résultat de notre recherche documentaire, une littérature très limitée est disponible sur le sujet, bien que plus de dix ans aient passé depuis le premier rapport. En résumant toutes les données disponibles, nous avons trouvé une relation positive entre le NRAMP1 D534N G> A et une SSR plus courte (P = 0.0002); en revanche, le rôle de hGPX1 Pro168Leu est largement indéfini (P = 0.81). L'analyse de sensibilité a détecté une évidente instabilité et il n'est pas clair si le biais de publication existe. Compte tenu de ces limitations, il est difficile de faire une description concluante.

## Conclusion

Très peu de données sont disponibles concernant l'association entre la récurrence tumorale et les polymorphismes de NRAMP1 D534N et hGPX1 Pro168Leu chez les patients NMIBC. Le NRAMP1 D534N pourrait augmenter le risque de récurrence, mais l'effet du hGPX1 Pro168Leu n'est pas clair. Il serait souhaitable qu'une enquête plus approfondie soit faite sur une taille d'échantillon plus élevée afin de mieux expliquer ce phénomène.

### Etat des connaissances actuelles sur le sujet

- La récurrence tumorale est fréquemment observée chez des patients NMIBC ;
- Des études antérieures ont fait preuves de résultats incohérents concernant la relation entre la récurrence et les polymorphismes génétiques de hGPX1 et NRAMP1 chez les patients NMBIC.

### Contribution de notre étude à la connaissance

- Très peu de données disponibles;
- Le NRAMP1 D534N pourrait augmenter le risque de récurrence, mais l'effet du hGPX1 Pro168Leu n'est pas clair;
- Il serait souhaitable qu'une enquête plus approfondie soit faite sur une taille d'échantillon plus élevée afin de mieux expliquer ce phénomène.

## Conflits d’intérêts

Les auteurs ne déclarent aucun conflit d'intérêts.
